# Items, Selections and Remarks

**Published:** 1870-01

**Authors:** W. W. Miner


### Items, Selections and Remarks.

BY W. W. MINER, A. B.

Dr. H. M. Beer, of Valparaiso, Ind., says in the *Philadelphia Reporter*, that a dose of two drachms chloroform, given internally, relieves an attack of fever and ague without the aid of quinine, and thinks the internal administration of chloroform as a hypnotic will be largely used in not this disease only, but in others dependent upon congestion and nervous irritation.—— Dr. Wm. McKean, of Mt. Hope, Ohio, reports that in place of a regular "Long tube Apparatus for Intestinal Obstructions" he has used a No. 12 flexible catheter in connection with a Davidson syringe, and that twice by this means he has effected the evacuation of obstructions which the strongest cathartics had failed to remove.—— Dr. A. J. Gardner, of Grand Rapids, Ohio, in the *Lancet and Observer*, recommends syrup of licorice as an excellent vehicle for the administration of quinine.

Dr. C. F. Chandler, chemist to the Metropolitan Board of Health, finds in his examinations of the kerosene oil of the New York market, that it contains almost without exception a large percentage of benzine and naptha. —— Two cases of death from the use of "Mrs. Winslow's Soothing Syrup," have been reported, and a correspondent of the *California Medical Gazette* reports of the second case that "the infant was six months old and had taken two doses of a teaspoonful each, within ten hours. An analysis of the syrup remaining in the phial, showed that it contained very nearly one grain of morphine and other alkaloids of opium to the ounce of syrup." The proprietors of the article deny the correctness of the analysis, but the *N. Y. Medical Journal* says that "We have seen many cases of infants who came into the Infants' Hospital here stupefied with this same soothing syrup." Another death attributed to the same cause is just reported in New Jersey.—Dr. A. T. Schutzer, of Baltimore, Md., relates the case of a lady 28 years of age, who has consumed in two years, five thousand eight hundred and forty ounces of laudanum, and spent eleven hundred and sixty dollars in purchasing it.—*Reporter.* —— A young man named Theodore Nicklos was arrested in Buffalo on suspicion of being the murderer of Dr. Andrew Mead, of Alleghany, and he has since confessed being the author of the crime.

Dr. Chas. Van Graefe, the celebrated German occulist, will visit the United States next March.—— J. Marion Sims, M. D., has returned from his European sojourn, and will hereafter make New York his permanent home.—*Lancet and Observer.* —— Dr. Thomas Addis Emmet has been elected a corresponding member of the Obstetrical Society of Berlin.—— The Homeopathic fraternity have opened a Hospital in New York City, wherein good results may be obtained if they pay their usual attention to hygienic management, and refrain from the system of over-dosing, which seems to be so prevalent among them now-a-days.—*N. Y. Medical Gazette.* —— Dr. Paul Schoeppe has obtained a writ of error. A recent suspicion that he was a Prussian forger has been disproven, and he is now awaiting executive clemency.

M. Albert, of Munich, has discovered a method by which a photographic negative is made to hold ink and give impressions after the manner of lithographic stone. —— M. L. Cailletet has published some experiments on the influence of pressure on chemical reaction. He shows that the action of hydrochloric acid on a sheet of zinc diminishes in exact proportion to the increase of atmospheric pressure, thus: when operating in open air, 10.0; at a pressure of 60 atmospheres, 4.7; at a pressure of 120 atmospheres, 0.1. Comparative experiments with nitric acid acting on a crystal of carbonate of lime, under a pressure of 150 atmospheres, and in the open air, show that the quantity of carbonate dissolved in an equal time is in the proportion of 1 to 15.09 —*American Exchange and Review.* —— Mr. James Young, the fortunate creator of the Paraffine industry, has given to the Andersonian University, at Glasgow, where the late Thomas Graham was a professor for a few years, the sum of 10,000 guineas to found a chair of applied chemistry, and it is somewhat remarkable that the person chosen to take the position is Mr. W. H. Perkins, who invented the first aniline that was made on a manufacturing scale, and who is the author of other important discoveries in the arts.—*Moniteur de la Teinture, Nashville Med. Journal*

Dr. H. O. Walker has retired from the editorship of the *Detroit Review of Medicine and Pharmacy*, and Drs. Geo. P. Andrews and A. B. Lyons succeed him in its publication. —— The *Medical News and Library* commences, this month, the publication in the journal *seriatim* of a work on "Diseases of the Spine and Nerves," by C. B. Radcliffe, M. D, F. R. C. P. —— The *Cincinnati Lancet and Observer* commence the new year with new type and headings, which present a quite tasteful appearance. —— The *Medical Bulletin* of Baltimore has changed the size of its page and shares in the general progressive movement. —— The *Richmond and Louisville Medical Journal* is giving short biographies of distinguished medical men, together with lithographic portraits of the same. In the December number of the journal we notice the face of the distinguished Dr. J. C. Warren, of Boston.

The medical classes of this city are about the same as last year; some increase, we believe, in the Jefferson School. In general, throughout the country, there is a diminished attendance. In Cincinnati the falling off from last year's number is 15 per cent. or more. We hear that in all the schools of St. Louis there are but one hundred students.—*Philadelphia Reporter.* —— The number of students in the class now attending lectures at the University of Buffalo is one hundred and thirteen, being an increase of twenty per cent over that of last year.

We notice in the *American Booksellers' Guide*, among the books announced as to be published this month: Second revised edition of Flint on Diagnosis, Pathology and Treatment of Disease of the Heart, Obstetric Aphorisms, an English reprint; H. C. Lea: A Text Book of Surgery by Prof. Frank H. Hamilton, a Text Book on Obstetrics by Prof. Wm. H. Byford, Holmes Surgery, Vol. II., On Epilepsy by M. Gonzalez Echeverria, M. D., Electricity and Electro-Therapeutics by Drs. Beard and Rockwell; Wm. Wood & Co.: Manuel of Hypodermic Medication by Robert Bartholow; J. B. Lippincott & Co. —— In the same we read: "A new illustrated magazine will shortly appear in New York City. It will be devoted to popular and original information for the people, and will fill a place held by no other publication. Prof. J. Walter Scott, the President of the New York Medical University, will be editor-in-chief."

